# Understanding the Interactions between Soft Segments in Polyurethanes: Structural Synergies in Blends of Polyester and Polycarbonate Diol Polyols

**DOI:** 10.3390/polym15234494

**Published:** 2023-11-22

**Authors:** Yuliet Paez-Amieva, José Miguel Martín-Martínez

**Affiliations:** Adhesion and Adhesives Laboratory, University of Alicante, 03080 Alicante, Spain; yuliet.paez@ua.es

**Keywords:** polyester polyol, polycarbonate diol polyol, polyols blends, soft segments, ester–carbonate interactions, self-adhesion

## Abstract

There are no previous studies on the interactions between polyols of different nature as a model for understanding the interactions between soft segments in PUs. In this study, different blends of two polyols of different natures (polyester—PE, and polycarbonate diol—CD) and similar molecular weights were prepared and their structural, thermal, surface, viscoelastic, and self-adhesion properties were assessed. Different experimental techniques were used: infrared spectroscopy (ATR-IR), differential scanning calorimetry (DSC), X-ray diffraction, thermal gravimetric analysis (TGA), and plate–plate rheology. PE showed a larger number of structural repeating units and a higher number of polar groups than CD, but the carbonate–carbonate interactions in CD were stronger than the ester–ester interactions in PE. The blending of CD and PE imparted synergic structural properties, particularly in the blends containing less than 50 wt.% PE, they were associated with the disrupt of the carbonate–carbonate interactions in CD and the formation of new ester–carbonate and hydroxyl–carbonate interactions. CD + PE blends with less than 50 wt.% PE exhibited higher glass transition temperatures, a new diffraction peak at 2θ = 24°, one additional thermal degradation at 426–436 °C, and a less-steep decline of the storage moduli. Furthermore, the different interactions between the polyol chains in the blends were also evidenced on their surface properties, and all CD + PE blends showed self-adhesion properties which seemed related to the existence of ester–carbonate and carbonate–carbonate interactions.

## 1. Introduction

The raw materials in the synthesis of polyurethanes (PUs) are isocyanates, polyols, and chain extenders [1]. PUs can be considered random segmented copolymers made of soft segments (SSs) and hard segments (HSs). HSs are produced by reacting the isocyanate and the chain extender, while SSs are constituted by the polyol chains [2]. Due to thermodynamic incompatibility between HSs and SSs, a micro-phase separation is produced in PUs which causes a discrete micro (or nano) domain structure [3,4,5,6,7,8,9,10,11]. The extent of micro-phase separation in PUs depends on the molecular weight, the chemical nature, and the structure of the reactants, among others [12,13].

The properties of PUs with HSs content lower than 30% are mainly determined by the interactions between SSs, i.e., between the polyol chains. The most common polyols in the synthesis of PUs are polyesters, polyethers, and, less commonly, polycaprolactones and polycarbonate diol polyols. The chemical nature, structure, and molecular weight of the polyols have an important influence on the properties and performance of the PUs [14,15].

The polyether polyols are obtained via condensation of two alcohols with elimination of water [1], and their properties are determined primarily by the ether linkage. PUs made with polyether polyols show a high resistance to hydrolytic degradation, high resiliency, water vapor permeability, abrasion resistance, and flexibility at low temperatures [14], but their mechanical properties are poor [16].

The polyester polyols are obtained via polycondensation of esters obtained by reacting a carboxylic acid and an alcohol [1]. The hydrolysis stability of the ester linkage is clearly inferior to that of the ether linkage because the released acid group from the ester linkage exerts an auto-catalytic effect which is deleterious to the SSs of PUs. The increased hydrophobicity and the higher number of ester groups improve the resistance to hydrolytic degradation of the polyester polyols [14]. The aliphatic polyester polyols exhibit excellent tensile properties, chemical resistance, cut resistance, tear strength, and high temperature stability.

In order to improve the hydrolytic stability of PUs, they can be synthesized with polycaprolactone or polycarbonate diol polyols. Their superior hydrolytic resistance is due to their low moisture absorption and, for polycarbonate diol polyols, the generation of CO_2_ upon hydrolysis without producing acidic moieties. PUs made with polycaprolactone polyols show high chemical resistance, good properties in a wide range of low and high temperatures, impact resistance, cut and tear resistance, sliding and abrasion resistance, and excellent durability [15]. However, PUs made with polycarbonate diol polyols show the greatest hydrolytic resistance, as well as a balanced combination of high resistance to heat, weathering, and abrasion [17,18].

Structurally, the polycarbonate and co-polycarbonate diols are linear, aliphatic polyols with carbonate linkages, the carbonate linkage providing high stability. There are two main synthesis procedures of polycarbonate diol polyols: (i) reaction of a diol with either dimethyl carbonate or diphenyl carbonate in the presence of a catalyst (tetrabutoxy titanium, dibutyltin oxide), followed by removal of the excess of reactants and mono-alcohol (methanol or phenol) under reduced pressure [19]; (ii) reaction of CO_2_ with an epoxide [20]. The polycarbonate diol polyols are primary diols and exhibit good reactivity with isocyanates. Because of their high polarity and strong carbonate bond, PUs prepared with polycarbonate diol polyols show good mechanical properties and an important degree of micro-phase separation [16,21,22]. Kojio et al. [23] established that the mechanical properties of elastomeric PUs made with polycarbonate diols were determined by the restriction of the crystallization of SSs. On the other hand, PUs made with polycarbonate diol polyols show high crystallinity, which affects the interactions among the polymer chains [24,25].

García-Pacios et al. [25] synthesized waterborne polyurethane dispersions (PUDs) intended for coatings made with polyols of similar molecular weights but different natures (polyether, polyester, and polycarbonate diol). The PUD obtained with polyether or polyester polyol showed a high degree of phase separation between SSs and HSs, and the best performance was obtained with the PUD coating made with polycarbonate diol; this was attributed to the higher polarity of the carbonate groups which favored the formation of additional physical bonds between SSs with respect to those obtained with polyether or polyester polyol.

Jofre-Reche et al. [2] studied the influence of the carbonate–carbonate interactions on the structure and properties of PUDs which were synthesized with different polycarbonate diols randomly copolymerized with hexamethylene and pentamethylene (C6-C5), tetramethylene (C6-C4), and trimethylene (C6-C3). The copolymers showed two glass transition temperatures, and the one at higher temperature was ascribed to the interactions between carbonate groups. It was concluded that the properties of the PUDs were affected by the degree of phase separation between HSs and SSs, the interactions between the carbonate groups, and the existence of even or odd methylene units in the copolymer backbone.

No one single polyol imparts all desired properties to PUs, so the use of blends of polyols of different nature in their synthesis may provide balanced properties. Some previous studies [16,26,27,28,29,30] have shown the beneficial properties of waterborne polyurethanes (PUDs) synthesized with blends of polyols of different nature. Gündüz and Kisakürek [26] showed that the partial replacement of polyester by polyether triol in the synthesis of PUDs imparted higher hardness, superior impact resistance, and flexibility. Kwak et al. [27] have found that the PUDs made with blends of polyester and polyether polyols showed intermediate storage moduli with respect to the ones made with single polyols, but the glass transition temperature (T_g_) values were similar to the one made with polyester; this was ascribed to greater crystallization ability and stronger intermolecular forces. Meng et al. [28] synthesized PUDs with blends of ethylene oxide–propylene oxide copolymer and poly(tetramethylene ether glycol), and they exhibited excellent waterproof properties. Cakic et al. [29] prepared PUDs with blends of polycaprolactone and polyethylene glycol or polypropylene glycol and found a significant different influence on the degree of micro-phase separation and the extent of crystallization. In a later study [30], they studied the properties of PUDs made with blends of polypropylene glycol and polycarbonate diol and found that they showed improved thermal stability. On the other hand, some literature has shown the relevance of the composition of the soft segments to the structure and performance of PUs [31,32,33].

In previous work, Fuensanta et al. [16] synthesized PUDs with blends of polyester and polycarbonate diol polyols and found different degrees of phase separation when the content of polycarbonate diol polyol was lower or greater than 50 wt.%; furthermore, complex interactions between the two types of SSs were noticed. On the other hand, upon ageing, the PUDs made with blends containing more than 50 wt.% polycarbonate diol polyol showed excellent adhesion due to the interactions between the carbonate groups in SSs and the higher miscibility between the hard and soft domains.

These previous studies have shown that the blending of polyols of different nature imparted improved mechanical, thermal, surface, and adhesion properties to the PUs [13,16,25,34]. More precisely, our previous work in PUDs [2,16,25] has shown the existence of synergies when polyols of different natures were used in their syntheses: they led to changes in the degree of micro-phase separation between HSs and SSs. Because the properties of PUs are strongly determined by the interactions between SSs, the interactions between the polyol chains of different nature would be the ultimate factor responsible for their different degree of phase separation. However, to the best of our knowledge, no previous studies on the interactions between polyols of different nature have been carried out, and they were considered in this study as a model for the interactions between SSs in PUs and PUDs. The assessment of those interactions will help to understand the structural features of PUs and PUDs made with blends of polyols. Therefore, in this study, different blends of polyester and polycarbonate diol polyols of similar molecular weights were prepared, and their structural, thermal, surface, viscoelastic, and self-adhesion properties were assessed.

## 2. Materials and Methods

### 2.1. Materials

The raw materials were polycarbonate of 1,6 hexanediol polyol with a molecular weight of 1000 Da–CD (Eternacoll^®^ UH-100, UBE Chemical Europe S.A., Castellón, Spain) and polyester polyol derived from 1,6 hexanediol with a molecular weight of 1000 Da–PE (Hoopol^®^ F-523, Synthesia Española, Barcelona, Spain).

### 2.2. Methods

#### 2.2.1. Preparation of the Polyols Blends

The polyols were dried under vacuum at 80 °C for 2 h. The amount of each polyol necessary to prepare a 5 g blend was added to a 15 mL polypropylene bottle (Hauschild SpeedMixer^®^, Hamm, Germany) maintained at 80 °C. The blend was stirred at 80 °C in a double centrifuge SpeedMixer DAC 150.1 FVZ-K system (FlackTek Inc., Landrum, SC, USA) at 2400 rpm for 1 min. Afterward, the blends of polyols were cooled down to room temperature. Before characterization, the polyols and the blends were preheated at 80 °C for 10 min to remove their thermal history.

The nomenclature of the polyol blends was “xCDyPE” (x and y are the percentages by weight of each polyol). Thus, 2CD8PE corresponds to the blend made with 20 wt.% polycarbonate diol polyol and 80 wt.% polyester polyol.

#### 2.2.2. Experimental Techniques

Infrared spectroscopy in attenuated total reflectance mode (ATR-IR spectroscopy): The chemical composition and the structure of the single polyols and their blends were assessed with ATR-IR spectroscopy. ATR-IR spectra were obtained in an Alpha spectrometer (Bruker Optik GmbH, Ettlingen, Germany) using a germanium prism. A total of 60 scans were performed with a resolution of 4 cm^−1^.

Differential scanning calorimetry (DSC): The structure and thermal properties of the single polyols and their blends were assessed with DSC under a nitrogen atmosphere (flow rate: 100 mL/min). Two consecutive thermal runs were performed in a DSC Q100 system (TA Instruments, New Castle, DE, USA): (i) heating from −80 °C to 200 °C (heating rate = 10 °C/min); (ii) cooling from 200 °C to −80 °C (cooling rate = 10 °C/min).

X-ray diffraction (XRD): The crystallinity of the single polyols and their blends was assessed with wide-angle X-ray diffraction in a Bruker D8-Advance system (Bruker, Etlinger, Germany) provided with a nickel filter and a Göebel mirror. A Kritalloflex K 760-80F X-ray generator (power: 3000 W; voltage: 20–60 kV; current: 5–80 mA) and the wavelength of copper (λ = 1.5406 Å) were used. A scan of 2θ angles from 5° to 90° was performed by varying 0.05° every 3 s.

Thermal gravimetric analysis (TGA): The structure and thermal properties of the single polyols and their blends were also assessed with TGA in a TGA Q500 system (TA Instruments, New Castle, DE, USA) under a nitrogen atmosphere (flow rate: 50 mL/min). A 9–10 mg sample was placed in a platinum crucible and heated from 35 °C to 600 °C using a heating rate of 10 °C/min. For removing char, at 600 °C, the nitrogen atmosphere was changed to air and heated for 15 min.

X-ray photoelectron spectroscopy (XPS): The chemical composition and the chemical species on the surfaces of the single polyols and their blends were assessed with XPS in a K-ALPHA instrument (Thermo Fisher Scientific, Waltham, MA, USA). The following experimental conditions were used: aluminum kα radiation (1486.6 eV), twin crystal monochromator, current of 3 mA and voltage of 12 kV. A spot of 400 µm diameter was analyzed, and a hemispherical analyzer operating in the constant energy mode was used. Charge compensation was achieved with the system flood gun that provides low energy electrons and low energy argon ions from a single source. Survey scans with pass energies of 200 eV were obtained and high resolution C1s and O1s XPS spectra were obtained with pass energies of 50 eV.

Ethylene glycol contact angle measurements: The contact angle measurements on the surfaces of the single polyols and their blends were carried out at 21 °C in an ILMS goniometer (GBX Instruments, Bourg de Pèage, France) by using ethylene glycol as the test liquid (purity > 99.0%, Merck-Schuchardt, Hohenbrunn, Germany). Ethylene glycol droplets of 3 μL were placed in different locations on the sample surface and measured 15 s after ethylene glycol drop deposition. The contact angles were the average of at least three drops placed on different zones of the surface with an error less than ± 2°.

Plate–plate rheology: The viscoelastic properties of the single polyols and their blends were assessed with plate–plate rheology in a DHR-2 rheometer (TA Instruments, New Castle, DE, USA). The samples were placed and melted at 80 °C on the lower stainless-steel plate, and an upper stainless-steel plate of 20 mm diameter was used. The gap was set to 0.40 mm and a frequency of 1 Hz was used. Temperature sweep tests were carried out by cooling down from 80 °C to −20 °C by using a cooling rate of 5 °C/min.

**Figure 1:**
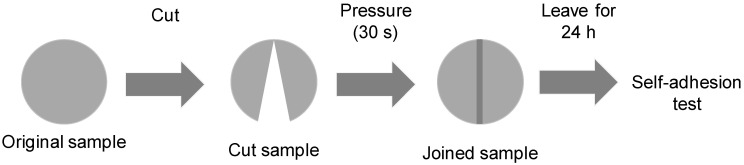
Assessment of the self-adhesion of the polyols and CD + PE blends.

Self-adhesion test: Cylindrical samples of the single polyols and their blends of 23.5 mm diameter and 5.6 mm height were prepared. The sample was cut down the middle with a scalpel (Figure 1). Subsequently, the cut parts were smoothly pressed together by hand at room temperature for 30 s and left to rest for 24 h. Then, the joined samples dropped several times from a height of 50 cm, observing if they de-bonded or not. The procedure is shown in Video S1 (Supplemental Materials file) for CD.

## 3. Results and Discussion

The properties of PUs with HSs content lower than 30% are mainly determined by physical interactions between SSs and the degree of micro-phase separation. The interactions among SSs depend mainly on the nature, molecular weight, and structure of the polyol.

The polyester polyols imparted good mechanical properties to the PUs but poor resistance to hydrolytic degradation [15,25]. In order to balance the hydrolytic degradation and the mechanical properties of the PUs, different blends of polyols of the same nature with different molecular weights [2,24,35] or polyols of different nature [16,25,26,27,28,29,30] have been used. The improved properties of PUs made with blends of polyols have been demonstrated, but poor attention has been paid to the interactions between SSs. It is our hypothesis that the interactions between SSs (i.e., the interactions among polyols of different nature) are the primary mechanism by which balanced and/or synergic properties are imparted to PUs. Therefore, in this study, the structural properties of different blends of polyester (PE) and polycarbonate diol (CD) polyols with similar molecular weights (1000 Da) are assessed. The structural features of the single polyols and their blends were evaluated with IR spectroscopy, DSC, wide-angle X-ray diffraction, TGA, XPS, contact angle measurements, and plate–plate rheology.

**Figure 2:**
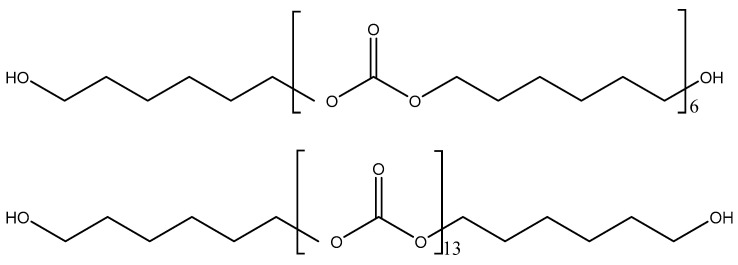
Chemical structure of CD (polycarbonate diol polyol).

**Figure 3:**
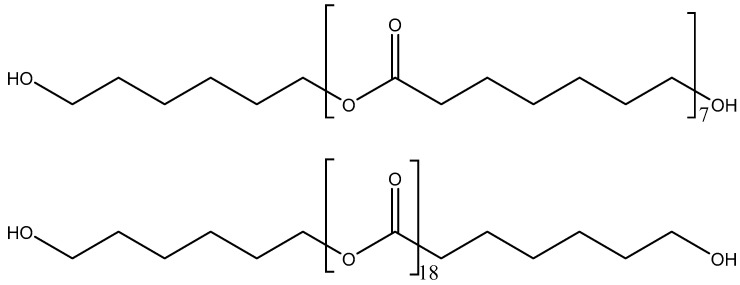
Chemical structure of PE (polyester polyol).

The structures of CD and PE (single polyols) are shown in Figure 2 and Figure 3, respectively. Considering that the molecular weight of both polyols is 1000 Da, there are six repeating carbonates of 1,6 hexanediol units in CD and seven repeating esters of 1,6 hexanediol units in PE, i.e., PE has a larger number of structural repeating units than CD. In addition, there are 13 carbonate groups in CD and 18 ester groups in PE, and, therefore, a higher number of dipole–dipole interactions between polar groups are produced in PE than in CD. However, the carbonate–carbonate interactions in CD are stronger than the ester–ester interactions in PE. On the other hand, both CD and PE show two end primary hydroxyl groups able to interact via hydrogen bond between themselves and with the carbonate or ester groups in the polyols. Furthermore, stronger hydroxyl–carbonate hydrogen bonds in CD than hydroxyl–ester hydrogen bonds in PE can be anticipated.

**Figure 4:**
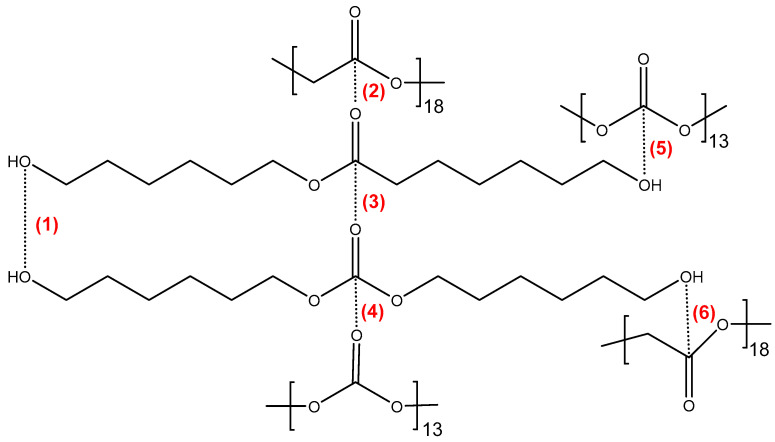
Potential interactions between polar groups in CD + PE blends: **(1)** hydroxyl–hydroxyl; **(2)** ester–ester; **(3)** ester–carbonate; **(4)** carbonate–carbonate; **(5)** hydroxyl–carbonate; and **(6)** hydroxyl–ester.

In CD + PE blends, ester–ester, carbonate–carbonate, ester–carbonate, hydroxyl–ester, and hydroxyl–carbonate interactions (Figure 4) can be produced; their number and strength will differ depending on the percentage of each polyol in the blend, and they may be different than the ones in the parent polyols.

**Figure 5:**
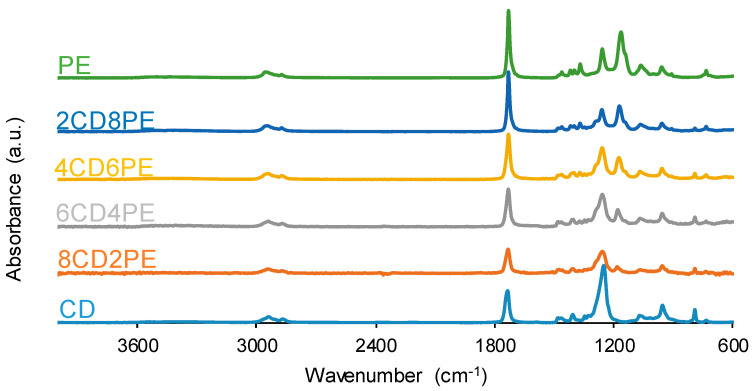
ATR-IR spectra of the polyols and CD + PE blends.

The chemical structure of the single polyols and their blends was assessed with ATR-IR spectroscopy. Figure 5 shows the ATR-IR spectra of CD, PE, and CD + PE blends, and the assignment of their most characteristic IR bands are given in Tables S1–S3 of the Supplementary Materials file. All ATR-IR spectra show the same absorption bands; the main difference is seen in the wavenumber of the OCC bands (1246–1257 for carbonate and ester groups, and 1171 cm^−1^ for ester group). The most intense IR bands correspond to C=O stretching at 1729–1735 cm^−1^, OC(O)O stretching at 1246–1257 cm^−1^, and OCC stretching at 1171 cm^−1^. The C=O stretching band appears at 1735 cm^−1^ in the ATR-IR spectrum of CD and displaces to lower wavenumbers in 2CD8PE and 4CD6PE; for CD + PE blends containing more than 50 wt.% PE, the C=O stretching band appears at 1730 cm^−1^, the same wavenumber as found in the ATR-IR spectrum of PE (Table S4 of the Supplementary Materials file). On the other hand, the OC(O)O stretching band of the carbonate group appears at 1250 cm^−1^ in the ATR-IR spectrum of CD and displaces to higher wavenumbers in the spectra of all blends. Therefore, different interactions between carbonyl groups are produced in CD + PE blends with respect to the parent polyols.

**Figure 6:**
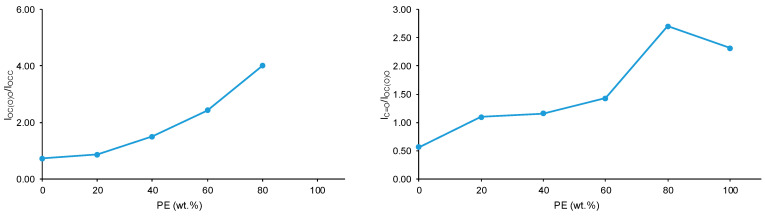
Variation of the I_OC(O)O_/I_OCC_ (**left**) and I_C=O_/I_OC(O)O_ (**right**) ratios of CD + PE blends as a function of PE amount.

The number of ester groups in PE is higher than that of carbonate groups in CD—Figure 2 and Figure 3. Because the ratio of the intensities of the C=O band with respect to that of the OC(O)O band—I_C=O_/I_OC(O)O_—is lower in the ATR-IR spectrum of CD than in that of PE (Figure 6), stronger interactions between the carbonate groups in CD with respect to the ones of the ester groups in PE are evidenced. The I_C=O_/I_OC(O)O_ ratio is higher in the ATR-IR spectra of CD + PE blends than in CD, more markedly in the blends with more than 50 wt.% PE, and the I_C=O_/I_OC(O)O_ ratio in 2CD8PE is even higher than the one in PE (Figure 6). Therefore, the interactions between the ester and carbonate groups in CD + PE blends differ with respect to the ones in PE and CD, and they depend on their PE content. On the other hand, the ratio of the intensities of the OC(O)O band (common to PE and CD) with respect to that of the OCC band (only in PE)—I_OC(O)O_/I_OCC_—in the ATR-IR spectra of CD + PE blends increases sharply when the amount of PE is higher than 50 wt.% (Figure 6). 2CD8PE and PE show almost equal I_OC(O)O_/I_OCC_ ratios because similar interactions between ester groups are produced in both. On the other hand, the variation of the I_C=O_/I_OC(O)O_ and I_OC(O)O_/I_OCC_ ratios as a function of PE amount is not linear (Figure 6), this indicates different interactions between CD and PE chains in the different blends with respect to the parent polyols.

**Figure 7:**
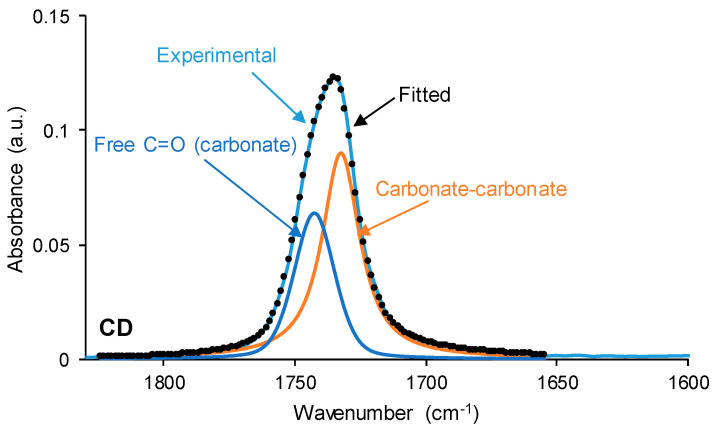

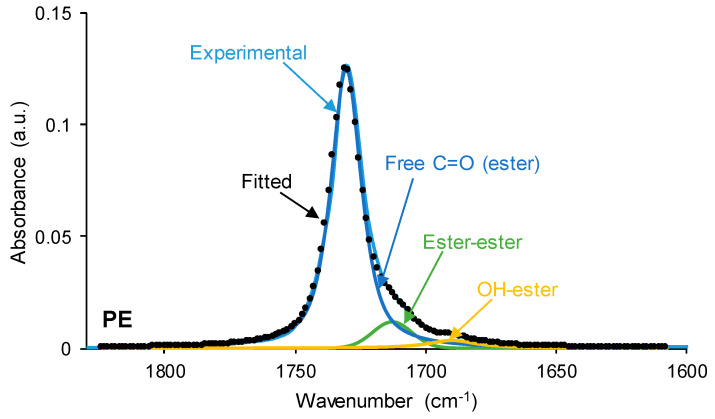
Curve fitting of the carbonyl stretching region of the ATR-IR spectra of CD and PE.

The interactions between the polar groups in the single polyols and their blends can be better evidenced through curve fitting of the carbonyl stretching region of the ATR-IR spectra. In this study, the curve fitting of the C=O stretching band was carried out by adjusting to the Gaussian function. The curve fitting of the carbonyl region of CD (Figure 7) shows 36% free C=O of the carbonate group at 1741 cm^−1^ and 64% bonded by dipole–dipole interactions C=O groups at 1730 cm^−1^, this confirming a strong interaction between the carbonate groups. The assignment of these groups is in agreement with the study by Niemczyk et al. [8], who reported three different contributions to the carbonyl region in PU made with polycarbonate diol polyol: (i) free carbonyl groups at 1744 cm^−1^; (ii) carbonyl groups bonded by dipole–dipole interactions at 1731 cm^−1^; and (iii) hydrogen bonds between the OH group of the polyol and C=O of the carbonate at 1719 cm^−1^. On the other hand, the curve fitting of the carbonyl region of PE (Figure 7) shows 88% free C=O of the ester group at 1730 cm^−1^, 8% bonded by dipole–dipole interactions C=O groups at 1712 cm^−1^, and 4% hydrogen-bonded OH-ester groups at 1689 cm^−1^. Thus, most ester groups in PE are free and a few hydrogen bonds between the terminal hydroxyl groups and the ester groups are produced. In a previous study [36], the existence of two contributions in the C=O region in hyperbranched polyester at 1741 cm^−1^ (free carbonyl) and 1728 cm^−1^ (OH-ester hydrogen bond) were reported.

**Figure 8:**
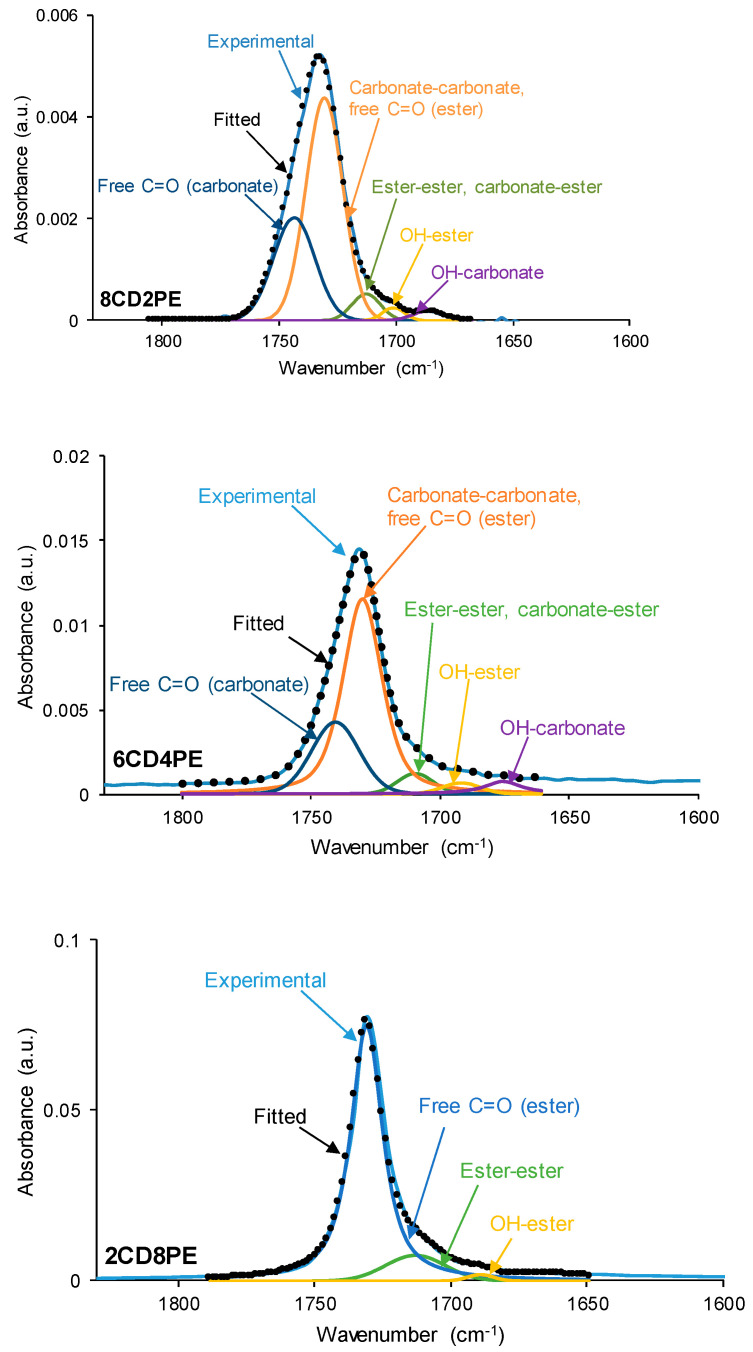
Curve fitting of the carbonyl stretching region of the ATR-IR spectra of some CD + PE blends.

The curve fitting of the carbonyl stretching region of the ATR-IR spectra of the CD + PE blends are shown in Figure 8 and Figure S1 of the Supplementary Materials file. All CD + PE blends show five contributions to the carbonyl region (2CD8PE is an exception because the same three contributions as in PE are distinguished) (Table 1); the one at 1675–1689 cm^−1^ is ascribed to hydroxyl–carbonate interactions. The wavenumber at which each contribution appears varies depending on the amount of PE in the blend (Table S4 of the Supplementary Materials file); this evidences the existence of different interactions in CD + PE blends with respect to the parent polyols. Thus, the addition of 20 wt.% PE to CD (8CD2PE) decreases the percentages of free carbonate and carbonate–carbonate interactions, and new contributions due to carbonate-ester, hydroxyl–ester, and hydroxyl–carbonate interactions can be distinguished (Table 1); these interactions differ from the ones in CD and PE. The same contributions to the carbonyl region appear in 6CD4PE, but a lower percentage of free carbonate groups and more important contributions of carbonate–carbonate, hydroxyl–ester, and hydroxyl–carbonate interactions are obtained (Table 1); furthermore, these contributions appear at a lower wavenumber (Table S4 of the Supplementary Materials file), indicating more net interactions between the polar groups at the expense of carbonate–carbonate interactions. In 4CD6PE, the same five contributions to the carbonyl region as in 8CD2PE and 6CD4PE can be distinguished, but the percentages of carbonate–carbonate and hydroxyl–carbonate contributions are lower and those of ester–ester and hydroxyl–ester are higher (Table 1), and the wavenumber of the ester–ester contribution is higher (Table S4 of the Supplementary Materials file). However, the same contributions to the carbonyl stretching band are found in 2CD8PE and PE because the addition of 20 wt.% CD does not alter significantly the interactions between polar groups in PE. Therefore, the interactions between the carbonyl groups of CD and PE change in the blends, particularly in those containing less than 50 wt.% PE.

**Figure 9:**
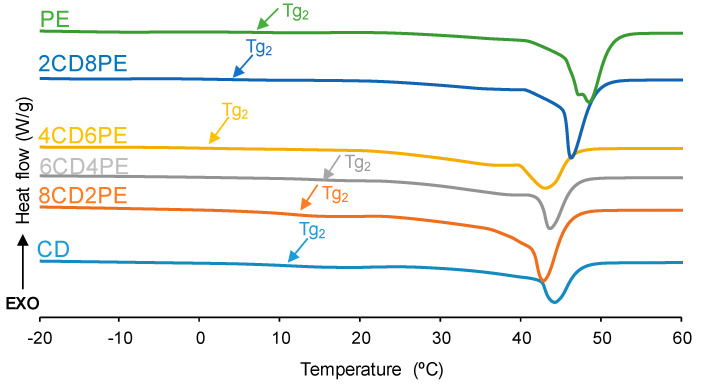
DSC curves of the polyols and CD + PE blends. First heating run.

The structural features of the polyols and CD + PE blends were also assessed with DSC. The DSC curves of the first heating run are given in Figure 9. All polyols and blends show two glass transition temperatures: T_g1_ (between −16 °C and −29 °C) due to van der Waals interactions between polyols chains; and T_g2_ (between 1 °C and 15 °C) due to ester–ester, ester–carbonate, and carbonate–carbonate interactions [2,21] (Figure S2 of the Supplementary Materials file). The highest T_g1_ and heat capacity at constant pressure (∆c_p1_) values correspond to CD and 8CD4PE due to more net interactions between the polyol chains (Table 2). The other blends show similar T_g1_ and ∆c_p1_ values because of similar interactions between the polymer chains; however, the ∆c_p1_ value in PE is lower than in the blends, indicating the existence of stronger interactions between ester and carbonate groups in CD + PE blends. On the other hand, the highest T_g2_ and ∆c_p2_ values are found in CD and the blends with less than 50 wt.% PE (11–15 °C); the increase in PE content causes lower T_g2_ and ∆c_p2_ values (Table 2). It should be noted that the ∆c_p1_ and ∆c_p2_ values of 8CD2PE are higher than the ones in CD, indicating the existence of ester–carbonate interactions in the blend. On the other hand, a melting temperature at 43–48 °C is evidenced in the DSC curves of the polyols and their blends. Because the melting enthalpy is higher in PE than in CD, the melting enthalpies of the blends increase with their increasing PE content (Table 2); however, the melting enthalpy of 8CD2PE is similar to that of PE (74 J/g), even though it contains 20 wt.% PE only. Therefore, the values of the different thermal events of the first DSC heating run in CD + PE blends are different than in the parent polyols, particularly in the blends containing less than 50 wt.% PE; this confirms the existence of additional interactions (mainly ester–carbonate interactions according to IR spectroscopy studies).

**Figure 10:**
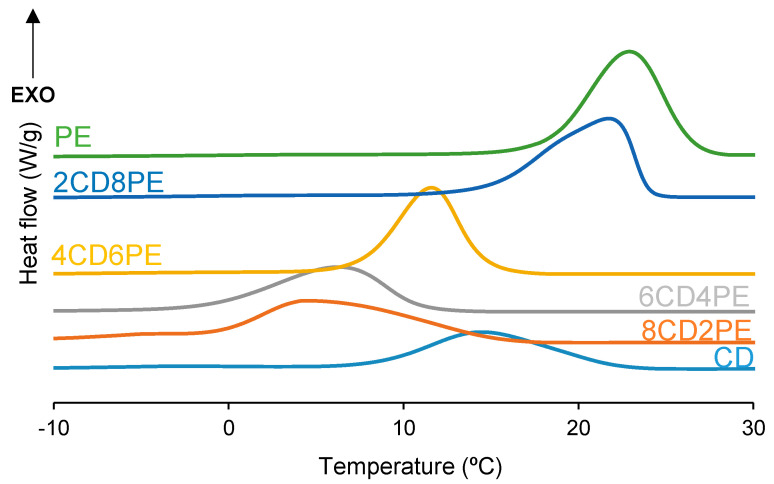
DSC curves of the polyols and CD + PE blends. Cooling run.

**Figure 11:**
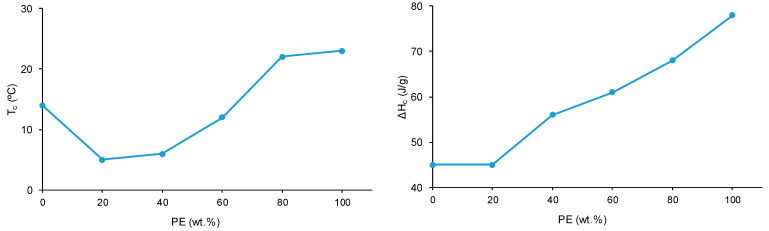
Variation of the crystallization temperature (**left**) and enthalpy (**right**) of CD + PE blends as a function of the amount of PE. Cooling DSC run.

After the first DSC heating run, the polyols and CD + PE blends were slowly cooled down to −80 °C, and one crystallization peak at 5–23 °C with crystallization enthalpies of 45–78 J/g was found (Figure 10). PE exhibits the highest temperature and enthalpy of crystallization and CD shows the lowest. Therefore, an increase in the temperature and enthalpy of crystallization in the CD + PE blends can be expected. While the crystallization enthalpy of the blends increases with their increasing PE content, except in 8CD2PE (Figure 11), the crystallization temperatures of the blends with less than 50 wt.% PE are lower (5–6 °C) than that of CD (14 °C), and higher and similar temperatures of crystallization are obtained in 2CD8PE and PE. Therefore, the interactions between PE and CD chains in the blends differ from the parent polyol, and they are stronger in the blends with less than 50 wt.% PE.

**Figure 12:**
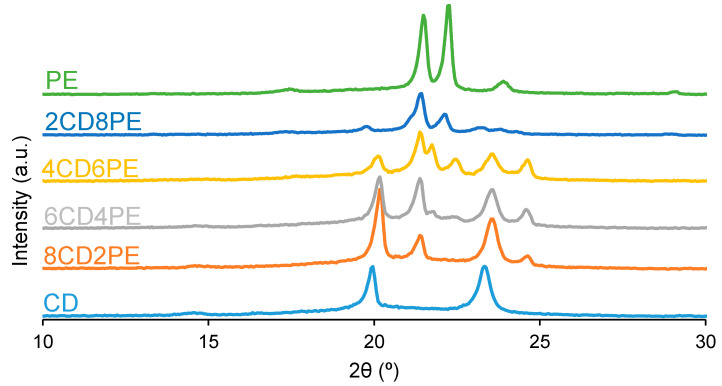
X-ray diffractograms of the polyols and CD + PE blends.

Wide-angle X-ray diffraction allows the assessment of the crystallinity of the polyols and CD + PE blends. The X-ray diffractogram of PE (Figure 12) shows two main intense diffraction peaks at 2θ values of 21° and 22°, and three additional low-intensity diffraction peaks at 2θ values of 17°, 23° and 29° can be distinguished. The X-ray diffractogram of CD (Figure 12) shows two main intense diffraction peaks at 2θ values of 19° and 23° (they are less intense than the ones of PE), and another low-intensity peak at a 2θ value of 14° also appears. Therefore, CD is less crystalline than PE, and the nature of the crystallinity is different in both polyols.

**Figure 13:**
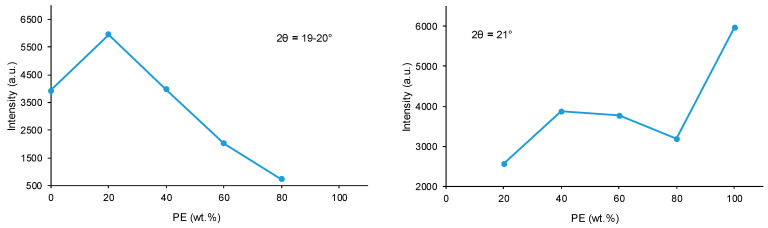
Variation of the intensity of the peaks at 2θ values of 19–20° (**left**) and 21° (**right**) of the CD + PE blends as a function of the amount of PE. X-ray diffractograms.

The X-ray diffractograms of the CD + PE blends show different peaks with different intensities depending on their PE content. The X-ray diffractogram of 8CD2PE shows four diffraction peaks at 2θ values of 20°, 21°, 23°, and 24° (Figure 12); the ones at 2θ values of 20° and 23° are the most intense. These two peaks can be ascribed to the interactions between CD chains. Because the peak at a 2θ value of 20° appears at 19° in CD and its intensity is higher in 8CD2PE, stronger and different interactions (likely ester–carbonate interactions) in 8CD2PE than in CD are evidenced (Figure 13). Therefore, the addition of only 20 wt.% PE disrupts the carbonate–carbonate interactions between CD chains, producing new interactions. In fact, while the peak at a 2θ value of 21° in 8CD2PE can be ascribed to ester–ester interactions, the one at a 2θ value of 24° does not appear in the parent polyols and can be ascribed to new ester–carbonate interactions. The X-ray diffractogram of 6CD4PE shows somewhat similar features than the one of 8CD2PE, but the intensity of the peak at 2θ = 21° (ester–ester interactions) is higher and the one at 2θ = 23° (carbonate–carbonate interactions) is lower (Figure 13). Furthermore, two small diffraction peaks at 2θ values of 21.3° and 22° can be distinguished; they do not appear in the parent polyols and correspond to new interactions.

The X-ray diffractogram of 4CD6PE (Figure 12) shows five intense peaks at 2θ values of 20°, 21°, 22°, 23° and 24°. The peaks at 2θ values of 21° and 22° can be ascribed to ester–ester interactions, and both are less intense than in PE. The peaks at 2θ values of 20° and 23° correspond to carbonate–carbonate interactions and they are much less intense than in CD. The peak at a 2θ value of 24° does not exist in the parent polyols; this could be an indication of the existence of ester–carbonate interactions. The X-ray diffractograms of 2CD8PE and PE are very similar, and they show two main diffraction peaks at 2θ values of 21° and 22° (Figure 12); however, the relative intensities of these two diffraction peaks are different, and additional low-intensity peaks at 2θ values of 20° and 23° due to CD appear in 2CD8PE.

Figure 13 shows the variation of the intensity of the diffraction peak at 2θ values of 19–20° in CD + PE blends; this peak only appears in the X-ray diffractogram of CD and can be attributed to carbonate–carbonate interactions. The addition of 20 wt.% PE only increases the intensity of the peak, and by further increasing the amount of PE in the blend, the intensity of the peak at 2θ = 19–20° decreases gradually. The intensity of the diffraction peak at a 2θ value of 21° due to PE (ester–ester interactions) is small in 8CD2PE and increases in the blend containing 40 wt.% PE (Figure 13); the blends containing 40–80 wt.% PE show similar intensity of the diffraction peak at 2θ = 21°, despite their very different PE content; this indicates the existence of similar interactions (likely ester–carbonate interactions).

**Figure 14:**
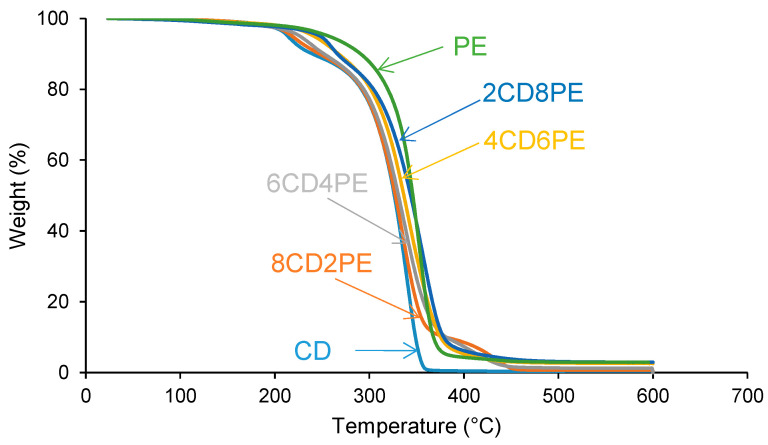
TGA curves of the polyols and CD + PE blends.

**Figure 15:**
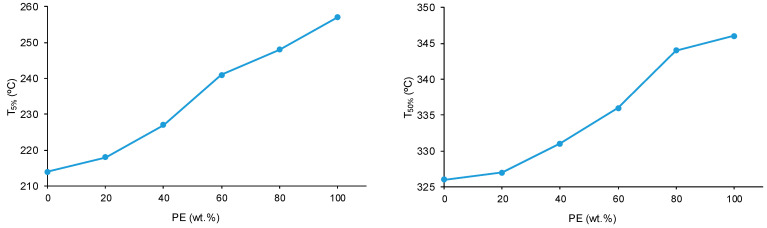
Variation of the temperature at which 5% (**left**) and 50% (**right**) mass loss are produced in CD + PE blends as a function of the amount of PE. TGA experiments.

The structural features of the polyols and CD + PE blends were also assessed with TGA. Figure 14 shows one main thermal degradation in PE starting at 257 °C and two thermal degradations in CD starting at 214 °C and 301 °C. The thermal degradation in CD starting at 214 °C is displaced to a higher temperature in the CD + PE blends; the displacement becomes more marked with increasing PE content. Furthermore, the TGA curves of 8CD2PE and 6CD4PE show an additional thermal degradation starting at 350 °C and 365 °C, respectively; this thermal degradation is not present in the TGA curves of the parent polyols nor in the blends with PE content higher than 50 wt.% and can be ascribed to ester–carbonate interactions. The differences in the thermal stabilities of the blends can be evidenced by the temperatures at which 5% (T_5%_) and 50% (T_50%_) mass loss are produced in the TGA curves. The T_5%_ and T_50%_ values of PE are higher than in CD, and the values of the blends are intermediate; the variation of the T_5%_ and T_50%_ values as a function of the PE content is not linear (Figure 15).

**Figure 16:**
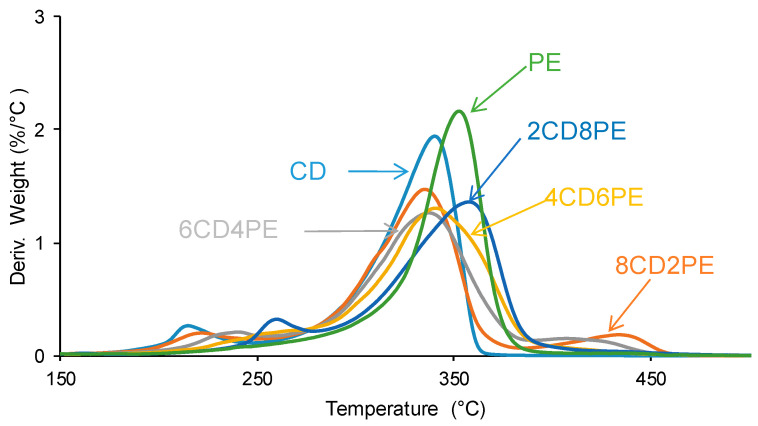
Derivative of the weight loss (DTGA) curves of the polyols and CD + PE blends. DTGA experiments.

**Figure 17:**
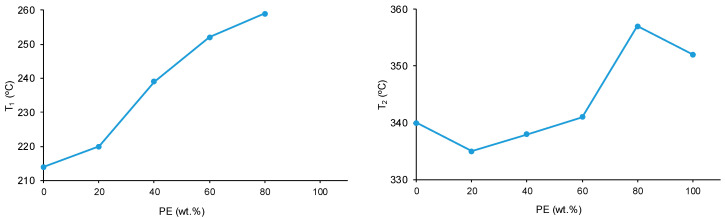
Variation of the degradation temperatures of CD + PE blends as a function of the amount of PE. DTGA experiments.

The differences in the TGA curves of the polyols and CD + PE blends can be better evidenced in the derivative of the TGA curves (DTGA curves) (Figure 16). The DTGA curve of CD shows two thermal degradations at 214 °C (likely due to carbonate–carbonate interactions) and 340 °C (likely due to the interactions between the chains); the main weight loss is produced at 340 °C (Table 3). The DTGA curve of PE shows one small degradation at 441 °C and one main thermal degradation at 352 °C; the higher thermal stability of PE can be ascribed to the higher number of polar groups than in CD. The DTGA curves of the blends with less than 50 wt.% PE show three thermal degradations at 220–229 °C, 335–338 °C, and 426–436 °C. The thermal degradations at 220–229 °C and 335–338 °C in the blends are also present in the DTGA curve of CD (Table 3), but they appear at higher temperatures—the higher the PE content, the higher the degradation temperature (Figure 17). Therefore, in the blends with less than 50 wt.% PE, some carbonate–carbonate interactions are substituted by ester-polycarbonate interactions. Furthermore, the appearance of a thermal degradation at 419–437 °C in all blends indicates the existence of novel interactions between the polyol chains, likely ester–carbonate interactions.

**Figure 18:**
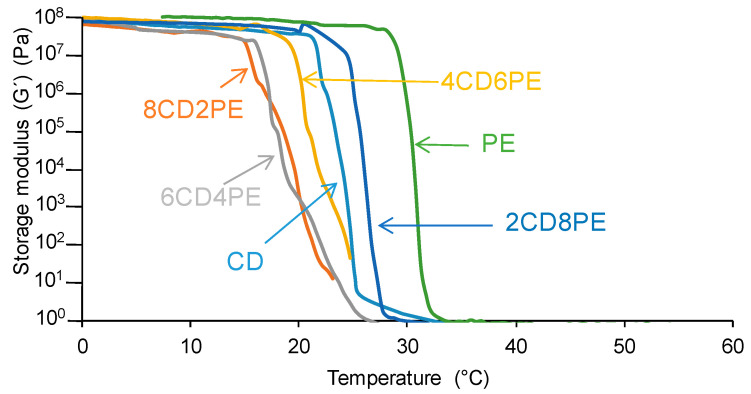
Variation of the storage (G′) moduli of the polyols and CD + PE blends as a function of the temperature. Plate–plate rheology experiments.

The structural features of the polyols and CD + PE blends may also affect their viscoelastic properties; as such, they were assessed with plate–plate rheology. Figure 18 shows the variation of the storage modulus (G’) as a function of the temperature for the polyols and their blends. In the glassy region, the storage moduli do not change with increasing temperature. At a given temperature, all polyols and blends show a sudden decrease in the storage moduli; this temperature differs in the polyols and CD + PE blends. The temperature at which G’ starts to decrease is significantly higher in PE (30 °C) than in CD (22 °C) because of the higher number of ester groups in PE than carbonate groups in CD; however, both show an abrupt decrease in G’ in a short temperature range. The addition of 80 wt.% PE produces a decrease in G’at an intermediate temperature between those of PE and CD; this is an indication of the disruption of some ester–ester interactions between the PE chains in 2CD8PE. However, the addition of 20–60 wt.% PE causes a decline in G’ at a lower temperature than in the parent polyols, and the decrease in G’ with the temperature is less steep; the higher the CD content, the more noticeable those changes. Therefore, in the blends containing 20–60 wt.% PE, the carbonate–carbonate interactions become less important and the existence of ester–carbonate interactions are evidenced by the less-steep decrease in G′ with temperature. Therefore, the different interactions in CD + PE blends containing less than 40 wt.% PE cause different viscoelastic properties.

**Figure 19:**
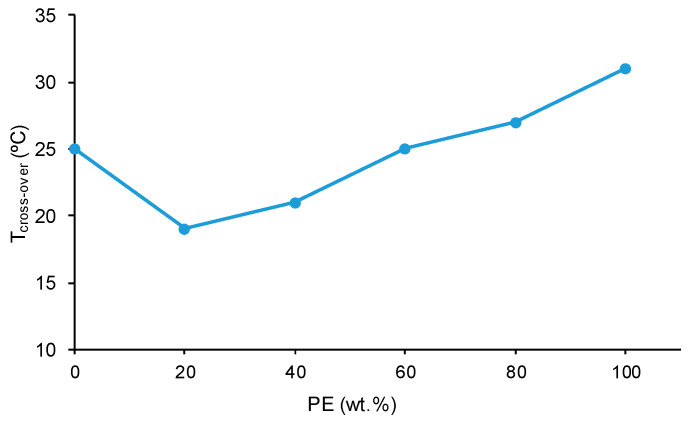
Variation of the temperature at the cross-over of the storage and loss moduli of CD + PE blends as a function of the amount of PE.

All polyols and blends show a cross-over of the storage (G′) and loss (G″) moduli (Figure S3 of the Supplementary Materials file). The temperature at the cross-over (T_cross-over_) is related to the interactions between the polymer chains; the higher the T_cross-over_ value, the greater the interactions. Figure 19 shows that PE has higher T_cross-over_ value than CD because of the higher number of ester–ester than carbonate–carbonate interactions. The addition of 20 wt.% PE decreases the T_cross-over_ value of CD because of the disruption of the carbonate–carbonate interactions, and the addition of higher amounts of PE gradually increases the T_cross-over_ value, likely due to the creation of ester–carbonate interactions.

The different interactions between the polyol chains in the CD + PE blends may also affect their surface properties; as such, they were assessed with XPS and contact angle measurements.

**Figure 20:**
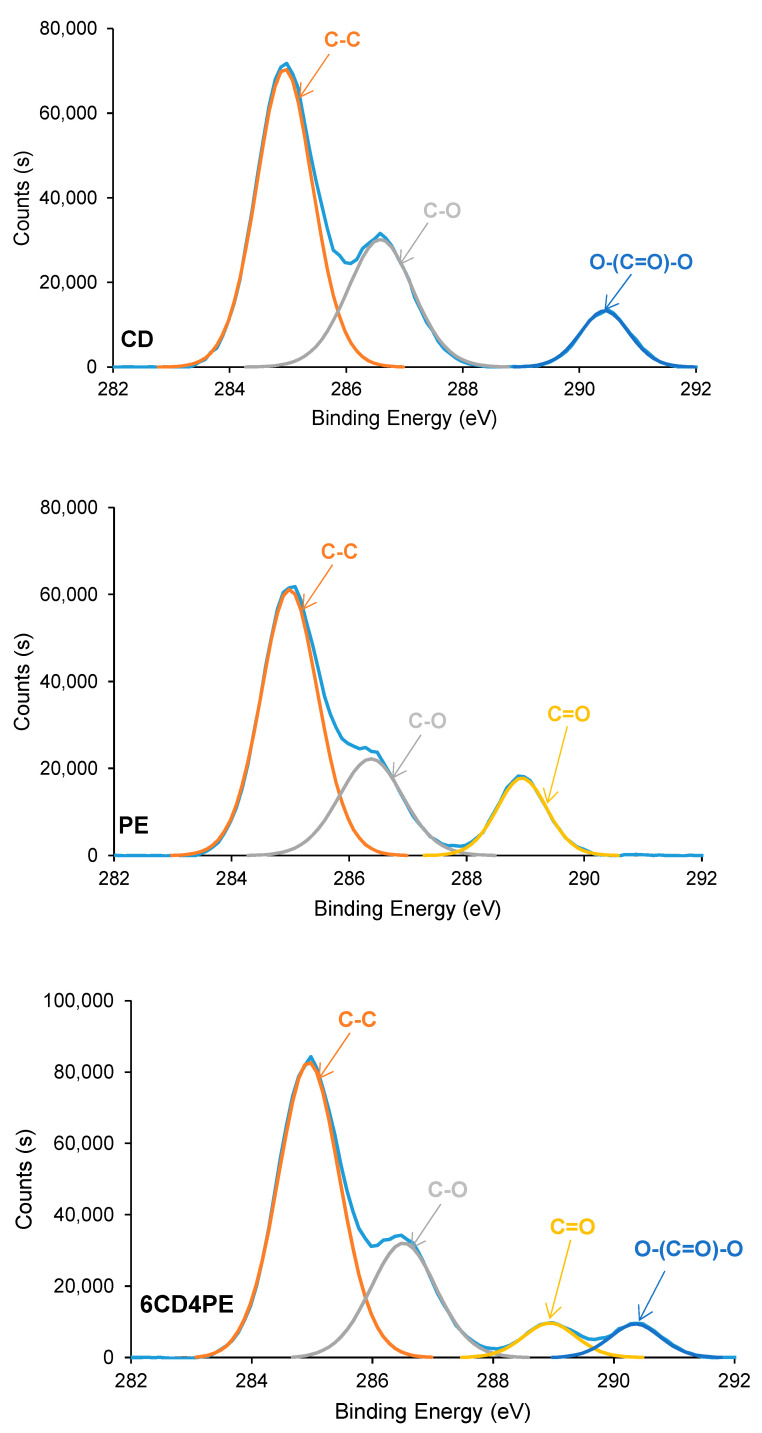
C1s photopeaks of CD, PE, and 6CD4PE surfaces.

The elemental composition on the surfaces of the polyols and CD + PE blends consists of 72–74 at.% carbon and 26–28 at.% oxygen; these are similar in all polyols and blends. The chemical species on the polyols and CD + PE surfaces were assessed using C1s high resolution XPS spectra. The C1s photopeak of CD (Figure 20) shows three different chemical species: 60 at.% C–C/C–H species at a binding energy of 285.0 eV; 30 at.% C–O species at a binding energy of 286.5 eV; and 10 at.% O–(C=O)–O species at a binding energy of 290.5 eV. This assignment was made according to XPS studies on polymers by Mishra et al. [37]. On the other hand, the C1s photopeak of PE (Figure 20) also shows three different chemical species: 60 at.% C–C/C–H species at a binding energy of 285.0 eV; 24 at.% C–O species at a binding energy of 286.5 eV; and 16 at.% C=O species at a binding energy of 289.0 eV. The C1s photopeaks of CD + PE blends show four different chemical species (C–C/C–H, C–O, C=O, and O–(C=O)–O); their percentages differ depending on their PE content (Figure 20 and Figure S4 of the Supplementary Materials file).

**Figure 21:**
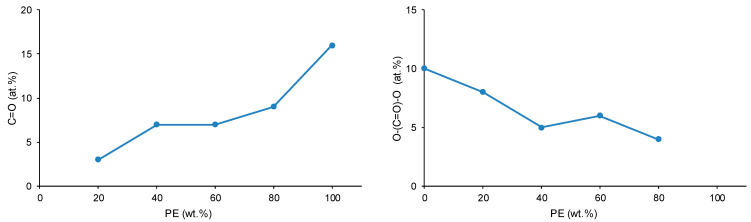
Variation of the C=O (**left**) and O–(C=O)–O (**right**) species on CD + PE blends’ surfaces as a function of PE content. C1s photopeak. XPS experiments.

The atomic percentages of the different chemical species on the CD + PE blends’ surfaces derived from the high resolution C1s photopeaks are shown in Table S5 of the Supplementary Materials file. The variations of the atomic percentages of the C=O and O–(C=O)–O species on the CD + PE blends’ surfaces as a function of their PE content are shown in Figure 21. The atomic percentage of the C–O species in the blends varies between 27 at.% and 31 at.%, and this species are also present on both parent polyols. The atomic percentage of the C=O species (only present on the PE surface) increases from 3 at.% to 7 at.% with an increase in the amount of PE from 20 wt.% to 40 wt%, and it does not change noticeably when adding more PE. Therefore, the interactions between the PE chains are somewhat similar on the surfaces of the blends containing 40–80 wt% PE, and they are significantly lower than on PE surface. On the other hand, the atomic percentage of the O–(C=O)–O species (only present on the CD surface) decreases gradually from 10 at.% to 4 at.% with an increase in the PE content in the blend; this is an indication of the disruption of the carbonate–carbonate interactions due to adding PE.

**Figure 22:**
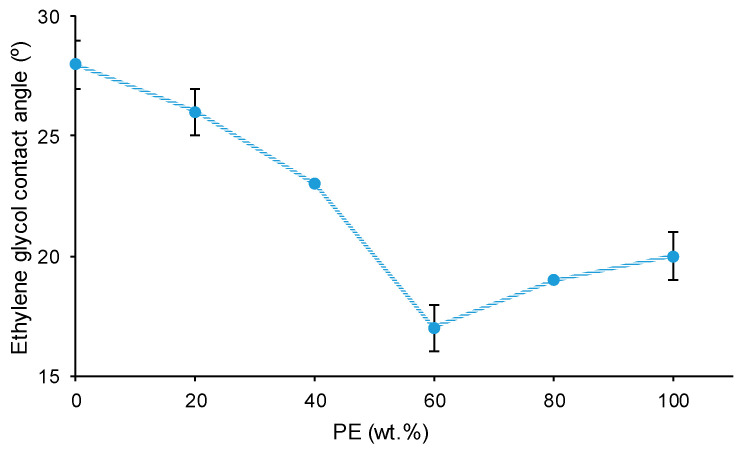
Variation of the ethylene glycol contact angle on polyols and CD + PE blends’ surfaces as a function of PE content.

The surface properties of the polyols and CD + PE blends were also assessed with ethylene glycol contact angles. Figure 22 shows a lower contact angle value on PE than on CD surface; this indicates better wettability. The lower contact angle on the PE surface can be ascribed to the existence of a larger number of polar groups than on the CD surface (Figure 2 and Figure 3). The contact angle values on the CD + PE blends’ surfaces with PE content lower than 60 wt.% PE decreases continuously; the decrease is more abrupt for the blends containing 40–60 wt.% PE. On the other hand, the contact angle values of the CD + PE blends’ surfaces containing more than 60 wt.% PE are somewhat similar. Therefore, the disruption of the carbonate–carbonate interactions in the blends’ surfaces as a result of adding up to 60 wt% PE is confirmed through ethylene glycol contact angle measurements; the increase in PE content above 60 wt.% in the blends does not markedly affect the carbonate–carbonate interactions nor the ester–ester interactions on their surfaces.

**Figure 23:**
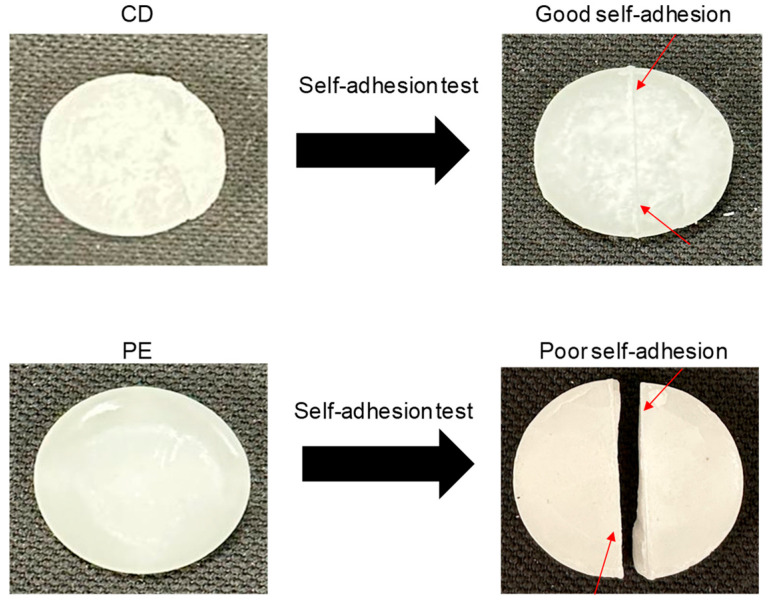
Self-adhesion test of CD and PE test samples. Red arrows indicate the location of the cut.

**Figure 24:**
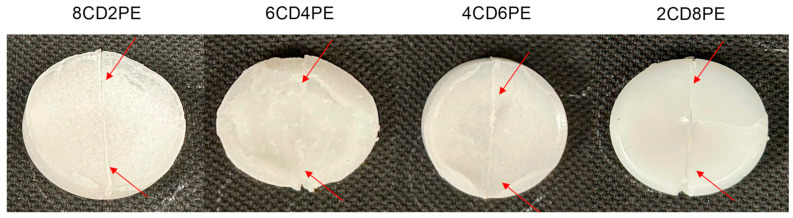
Appearance of the CD + PE test samples after self-adhesion test. Red arrows indicate the location of the cut.

The existence of ester–carbonate and carbonate–carbonate interactions may affect the adhesion property of the blends. The self-adhesion properties of the polyols and their blends were assessed by cutting the pieces by half and rejoining them for 30 s by hand under a mild pressure. After 24 h of joint formation, the joined samples were allowed to fall down several consecutive times from a high of 50 cm, observing whether they de-bonded or not. The results of the self-adhesion test of CD are shown in Video S1 of the Supplementary Materials file, in which is evidenced that, after seven consecutive falls, de-bonding is not produced. The results of the self-adhesion test of PE are shown in Video S2 of the Supplementary Materials file, in which is evidenced that, after six consecutive falls, de-bonding is produced. Figure 23 shows the appearance of the CD and PE samples before and after the self-adhesion test. The results of the self-adhesion of 2CD8PE are shown, as a typical example of the blends, in Video S3 of the Supplementary Materials file, in which is evidenced that, after eight consecutive falls, de-bonding is not produced. Figure 24 shows the appearance of the CD + PE blends samples after the self-adhesion test; all of them show self-adhesion properties after eight consecutive falls, likely due to the existence of ester–carbonate and carbonate–carbonate interactions

The original content of this manuscript was published on 20th October 2023 as a preprint in the Preprint.org platform [38].

## 4. Conclusions

The blending of polyols of different natures (PE and CD) and similar molecular weights changed the extent and the nature of the interaction between their polar groups, leading to synergic structural features in the blends containing less than 50 wt.% PE.

PE showed a larger number of structural repeating units and a higher number of polar groups than CD, but the carbonate–carbonate interactions in CD were stronger than the ester–ester interactions in PE; this caused a lower ethylene glycol contact angle on the PE surface and the existence of self-adhesion in CD. Because of the higher number of ester groups in PE than carbonate groups in CD, the temperature at which G’ decreased in the rheological curves and the temperature at the cross-over of G’ and G″ were higher in PE.

The blending of CD and PE imparted synergic structural properties, particularly in the blends containing less than 50 wt.% PE; they were associated with the disruption of carbonate–carbonate interactions and the formation of new ester–carbonate and hydroxyl–carbonate interactions. The blends showed five contributions to the carbonyl region, and the wavenumber at which each contribution appeared varied depending on the amount of PE. All polyols and blends showed two glass transition temperatures, T_g1_ (due van der Waals interactions between polyols chains) and T_g2_ (due to ester–ester, ester–carbonate, and carbonate–carbonate interactions). The X-ray diffractograms of the CD + PE blends showed a new peak at 2θ = 24° with respect to the parent polyols; this peak was ascribed to the ester–carbonate interactions. Furthermore, the CD + PE blends with less than 50 wt.% PE showed three thermal degradations; the one at 426–436 °C was associated with ester–carbonate interactions. On the other hand, all polyols and blends showed a crossover of the storage (G′) and loss (G″) moduli, and the temperature at the crossover in the blends containing 40 wt.% or more PE gradually increased due to the creation of ester–carbonate interactions.

The different interactions between the polyol chains in the CD + PE blends were also evidenced though their surface properties. The atomic percentage of C=O species (only present on PE surface) increased with an increase in the amount of PE from 20 wt.% to 40 wt%, and it did not change noticeably after adding more PE. For the atomic percentage of the O–(C=O)–O species (only present on the CD surface), the opposite trend occurred, indicating the disruption of the carbonate–carbonate interactions due to adding PE. On the other hand, the ethylene glycol contact angle values of the blends’ surfaces with PE content lower than 60 wt.% decreased continuously; this agreed with the disruption of the carbonate–carbonate interactions in the blends.

Finally, CD and all CD + PE blends showed self-adhesion properties, which seemed related to the existence of ester–carbonate and carbonate–carbonate interactions.

## Figures and Tables

**Table 1:** Percentages of C=O species in the carbonyl stretching region of the ATR-IR spectra of CD + PE blends. Curve fitting.

Wavenumber (cm^−1^)	Percentage (%)				Assignment
	8CD2PE	6CD4PE	4CD6PE	2CD8PE	
1675–1689	2	4	2	-	OH–carbonate
1691–1702	2	3	3	1	OH–ester
1709–1718	5	5	14	11	Carbonate–ester, ester–ester
1729–1730	60	64	54	88	Carbonate–carbonate, free C=O (ester)
1739–1743	31	24	27	-	Free C=O (carbonate)

**Table 2:** Thermal events in the DSC curves of the polyols and CD + PE blends. First heating run.

Polyol	T_g1_ (°C)	$\Delta cp_{1}\;(J/g$ °C)	T_g2_ (°C)	$\Delta cp_{2}\;(J/g$ °C)	T_m_ (°C)	ΔH_m_ (J/g)
CD	−16	0.34	11	0.58	44	40
8CD4PE	−16	0.43	12	0.97	43	74
6CD4PE	−27	0.18	15	0.29	43	55
4CD6PE	−27	0.19	1	0.17	43	70
2CD8PE	−29	0.21	4	0.16	46	70
PE	−28	0.14	7	0.10	48	74

**Table 3:** Temperatures and weight losses of the thermal degradations of the polyols and CD + PE blends. DTGA experiments.

Polyol	1st Degradation		2nd Degradation		3rd Degradation	
	T_1_ (°C)	Weight Loss_1_ (%)	T_2_ (°C)	Weight Loss_2_ (%)	T_3_ (°C)	Weight Loss_3_ (%)
CD	214	11	340	89	-	-
Y8CD4PE	220	11	335	80	436	9
Y6CD4PE	239	12	338	80	426	8
Y4CD6PE	252	13	341	81	437	3
Y2CD8PE	259	13	357	81	419	3
PE	-	-	352	96	441	4

## Data Availability

Data are contained within the article and supplementary materials.

## Supplementary Materials

The following supporting information can be downloaded at: https://www.mdpi.com/article/10.3390/polym15234494/s1, Figure S1: Curve fitting of the carbonyl stretching region of the ATR-IR spectrum of 4CD6PE; Figure S2: DSC curve of 4CD6PE. Region of the glass transition temperatures. First heating run; Figure S3: Variation of the storage (G′) and loss (G′′) moduli of CD as a function of the temperature; Figure S4: C1s photopeaks of different CD+ PE blends; Table S1: Assignment of the main absorption bands in the ATR-IR spectrum of CD; Table S2: Assignment of the main absorption bands in the ATR-IR spectrum of PE; Table S3: Assignment of the main absorption bands in the ATR-IR spectra of CD + PE blends; Table S4: Wavenumbers of species in the carbonyl stretching region of the ATR-IR spectra of the polyols and CD + PE blends; Table S5: Chemical species on the polyols and CD + PE blend surfaces. C1s photopeak. XPS experiments; Video S1: Self-adhesion test of CD; Video S2: Self-adhesion test of PE; Video S3: Self-adhesion test of 2CD8PE.
