# Supplementary material for: Understanding the Interactions between Soft Segments in Polyurethanes: Structural Synergies in Blends of Polyester and Polycarbonate Diol Polyols
Source: Polymers (Basel). 2023 Nov 22;15(23):4494. doi: 10.3390/polym15234494 (PMC10708524; doi:10.3390/polym15234494)
Supplement: Supplementary file 1 [file polymers-15-04494-s001.zip › 2023-11-22 Revised Supplementary Materials-keynoted.pdf]

Understanding the interactions between soft segments in polyurethanes : Structural synergies in blends of polyester and polycarbonate diol polyols

Yuliet Paez-Amieva and José Miguel Martín-Martínez

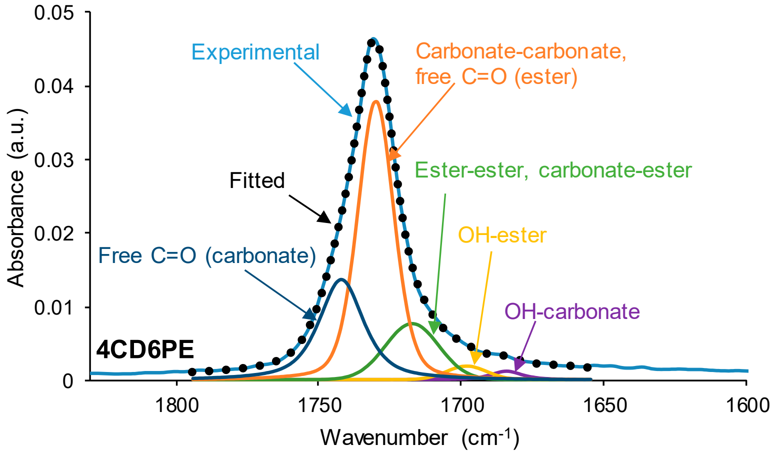

Figure S1. Curve fitting of the carbonyl stretching region of the ATR-IR spectrum of 4CD6PE.

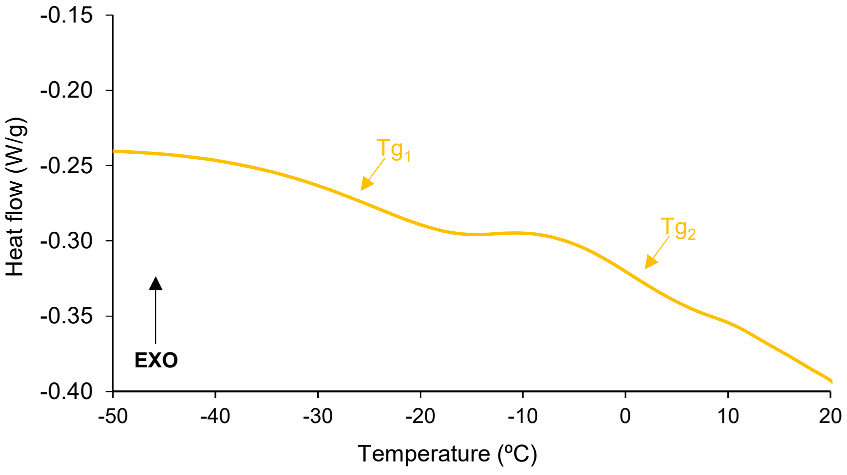

Figure S2. DSC curve of 4CD6PE. Region of the glass transition temperatures. First heating run.

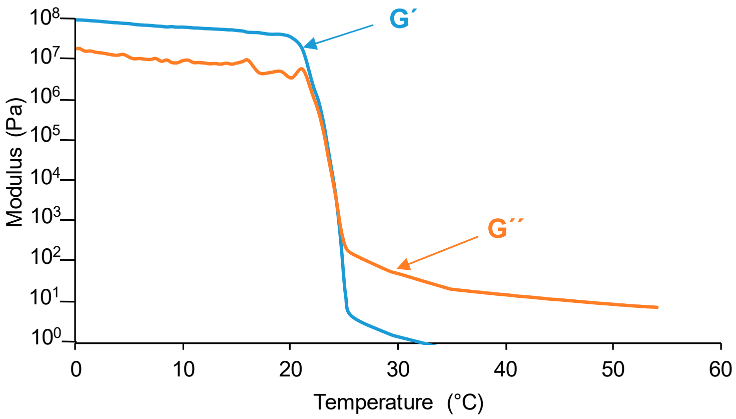

Figure S3. Variation of the storage (G´) and loss (G´´) moduli of CD as a function of the temperature.

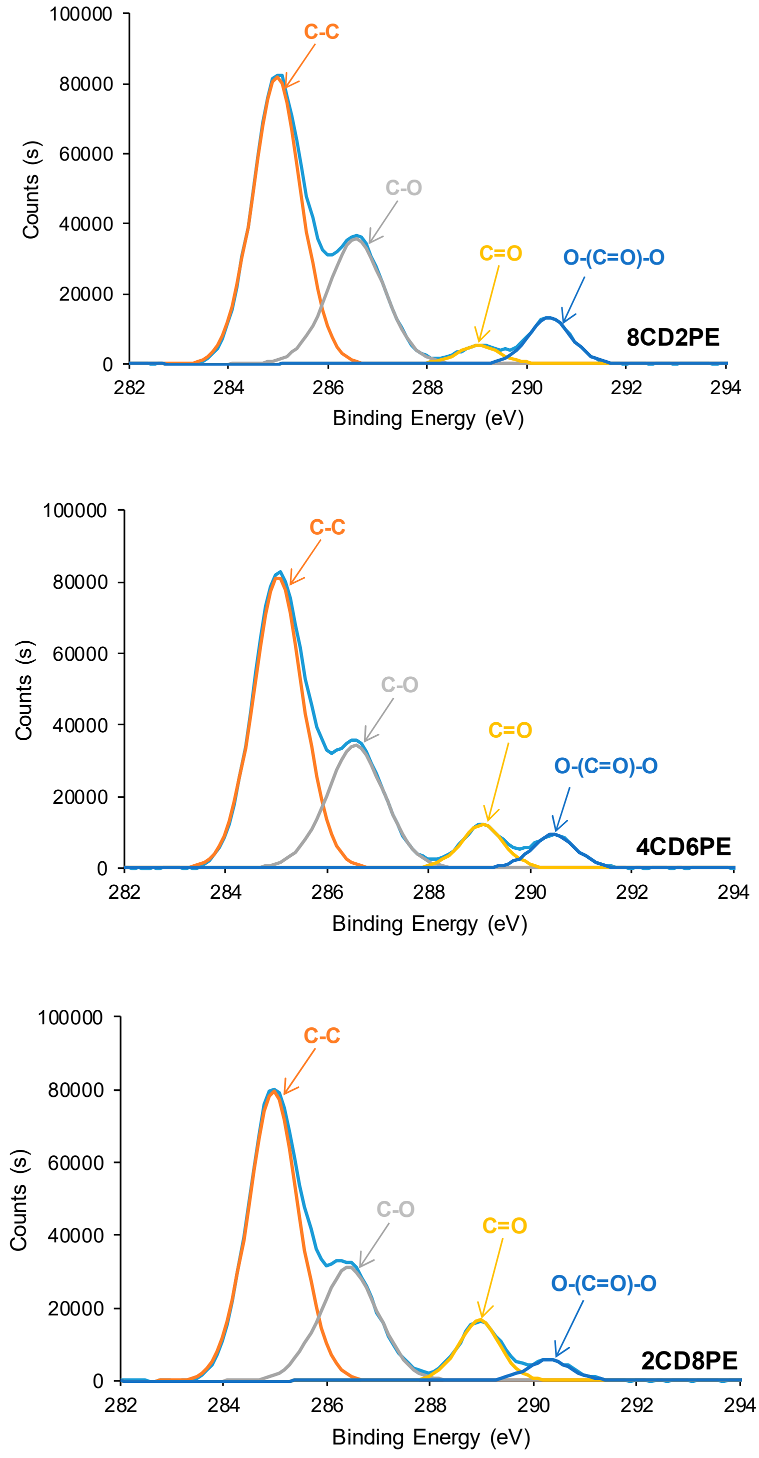

Figure S4. C1s photopeaks of different CD+PE blends´s surfaces.

Table S1. Assignment of the main absorption bands in the ATR-IR spectrum of CD.

Wavenumber (cm^{-1})	Assignment
3448	O-H stretching
2934, 2860	C-H stretching
1735	C=O stretching (carbonate)
1482, 1459	δ (asym) CH_2, δ CH_3
1405, 1343, 1326	δ (sym) CH_2
1250	OC(O)O stretching (carbonate)
1067, 1019, 954	C-O-C stretching (carbonate)
735	δ CH_2

Table S2. Assignment of the main absorption bands in the ATR-IR spectrum of PE.

Wavenumber (cm^{-1})	Assignment
3492	O-H stretching
2950, 2867	C-H stretching
1730	C=O stretching (ester)
1461	δ (asym) CH_2, δ CH_3
1367	δ (sym) CH_2
1257	CC(O)O stretching (ester)
1171	OCC stretching (ester)
1064, 994, 959, 905	C-O-C stretching
731	δ CH_2

Table S3. Assignment of the main absorption bands in the ATR-IR spectra of CD+PE blends.

Wavenumber (cm^{-1})	Assignment
3439-3465	O-H stretching
2937-2948; 2867	C-H stretching
1729-1732	C=O stretching (ester, carbonate)
1472-1480; 1458-1464	δ (asym) CH_2, δ CH_3
1397-1407; 1367-1375; 1346-1351	δ (sym) CH_2
1254-1257	CC(O)O stretching (ester, carbonate)
1171-1182	OCC stretching (ester)
1064-1066; 951-956	C-O-C stretching (ester, carbonate)
731-739	δ CH_2

Table S4. Wavenumbers of species in the carbonyl stretching region of the ATR-IR spectra of the polyols and CD+PE blends.

**Wavenumber (cm^{-1})**						**Assignment**
CD	8CD2PE	6CD4PE	4CD6PE	2CD8PE	PE	
-	1689	1675	1689	1689	1689	OH-ester
-	1701	1691	1702	-	-	OH-carbonate
-	1713	1709	1718	1712	1712	Carbonate-ester; ester-ester
1730	1730	1729	1730	1730	1730	Carbonate-carbonate; free C=O (ester)
1741	1743	1740	1739	-	-	Free C=O (carbonate)

Table S5. Chemical species on the polyols and CD+PE blends´ surfaces. C1s photopeak. XPS experiments.

**Polyol/Blend**	**C-C/C-H (at.%)**	**C-O (at.%)**	**C=O (at.%)**	**O-(C=O)-O (at.%)**
CD	60	30	-	10
8CD2PE	58	31	3	8
6CD4PE	62	26	7	5
4CD6PE	58	29	7	6
2CD8PE	60	27	9	4
PE	60	24	16	-
